# Orthokeratology in controlling myopia of children: a meta-analysis of randomized controlled trials

**DOI:** 10.1186/s12886-023-03175-x

**Published:** 2023-10-31

**Authors:** Xue Li, Meiling Xu, Shanshan San, Lanzheng Bian, Hui Li

**Affiliations:** Department of nursing, Children’s Hospital of Nanjing Medical University, No. 72, Guangzhou Road, Gulou District, Nanjing, China

**Keywords:** Orthokeratology, Myopia, Children, Care

## Abstract

**Background:**

Delaying the development and lowering the progression of myopia in children is the focus of current ophthalmology researches. We aimed to evaluate the role of orthokeratology in controlling myopia of children, to provide insights to the clinical treatment and care of children with myopia.

**Methods:**

Two investigators searched the The Cochrane Library, Embase, Pubmed, China national knowledge infrastructure, China biomedical literature database, WanFang and Weipu databases for randomized controlled trials(RCTs) on the role of orthokeratology in controlling myopia of children up to November 5, 2022. Two researchers independently searched, screened and extracted the studies according to the inclusion and exclusion standards. RevMan5.3 software was used for statistical analysis.

**Results:**

A total of 14 RCTs involving 2058 children were included in this meta-analysis. Synthesized outcomes indicated that orthokeratology improved the uncorrected visual acuity(MD = 0.40, 95%CI: 0.05 ~ 0.74), reduced the diopter change(MD=-3.19, 95%CI: -4.42~-1.95), changes of corneal curvature(MD=-3.21, 95%CI: -3.64~-2.79), the length of ocular axis (MD=-0.66, 95%CI: -1.27~-0.06) and amount of ocular axis change(MD=-0.42, 95%CI: -0.64~-0.21) after 1 year of wearing orthokeratology(all P < 0.05). Besides, orthokeratology reduced the diopter change (MD=-3.22, 95%CI: -4.86~-1.58), the length of ocular axis (MD=-1.15, 95%CI: -2.25~-0.06) and the amount of ocular axis change after 2 year of wearing orthokeratology (MD=-0.53, 95%CI: -0.96~-0.11) after 2 year of wearing orthokeratology (all P < 0.05). No publication biases were found amongst the synthesized outcomes (all P > 0.05).

**Conclusions:**

Orthokeratology delays the progression of myopia in children, the long-term effects of orthokeratology need further investigations in future studies.

## Background

Myopia is the refractive state in which the light of distant objects is focused in front of the retina when the eye is relaxed. It has become a global public health problem [1]. Myopia is affected by many factors, such as heredity and environment [2]. At present, it can only be controlled clinically. The commonly used methods to correct and prevent myopia include outdoor activities, drugs, wearing frame glasses, hard contact lenses and surgical treatment. In recent years, the control effect of orthokeratology on the development of myopia has been widely recognized. With the increasing number of users, the unique design of orthokeratology lenses and the way of night wear have gradually exposed clinical problems [3–5]. Due to the differences in research quality, there is still a lack of evaluation on myopia control effect of orthokeratology with different treatment duration.

It’s been reported that myopia in children is related to severe myopia in adulthood [6, 7]. It is important to control the development of myopia in school age children to reduce the incidence rate of severe myopia in the future [8–10]. There are many studies on the effectiveness of orthokeratology in controlling myopia, but there are differences in follow-up time, research design, research object, etc. This study aimed at these differences, and planned to systematically evaluate the researches on orthokeratology in controlling the development of myopia in school-age children, so as to evaluate the effectiveness of using orthokeratology in the myopia of children, and provide reliable evidences for the clinical treatment and nursing care of myopia in children.

## Methods

This meta-analysis and systematic review was conducted following the preferred reporting items for systematic reviews and meta-analyses (PRISMA) statement [11].

### Literature search

The databases searched in this meta-analysis included: The Cochrane Library, Embase, Pubmed, China national knowledge infrastructure, China biomedical literature database, WanFang and Weipu databases. The retrieval time limit was from the establishment of the database to November 5, 2022. Both the subject words and free words were used for literature search, the retrieval strategy was adjusted according to the specific database. The search strategies were as follows: (“orthokeratology” OR “orthokerological procedure” OR “procedure” OR “orthokerological” OR “procedures” OR “orthokerological” OR “orthokerological lens” OR “ortho-K lens” OR “OK lens” OR “reverse geometry lens”) AND (“Myopia” OR “nearsightedness” OR “Near sight” OR “short sight” OR “shortsightedness”). Besides, in order to include more related studies for this meta-analysis, the literatures of relevant reviews and references were searched manually.

### Inclusion and exclusion criteria

The inclusion criteria of this meta-analysis were as follows: Children with myopia whose age was 6–18 years old and whose spherical lens was less than − 6.00 D and cylindrical lens was less than − 1.5D, the follow-up period should be at least one year; the children underwent orthokeratology or frame mirror treatment; related outcomes were reported including axial length, corneal curvature, naked eye vision and diopter et al. the study design should be randomized controlled trial (RCT).

The exclusion criteria of this meta-analysis were as follows: reports including reviews, letters, case reports and comments were excluded; repeated published literature; the full text of literature could not be obtained.

### Literature screening and data extraction

Two researchers independently searched, screened, extracted and checked the documents according to the inclusion and exclusion standards. We removed the irrelevant documents by reading the title and abstract, and further read the full text of the retained documents to determine whether they were included. If there was any disagreement between the two researchers, a third researched was invited for discussion to obtain a consistent result.

The two authors extracted data from the original literature, including the author’s name, publication time, age, follow-up time, number of eyes, outcome indicators and research conclusions. All differences and disputes are resolved through discussion for reaching consensus.

### Quality assessment

The risk of bias of included RCTs as evaluated using the Cochrane risk of bias assessment instrument [12] by two authors. The bias has been evaluated across four domains: random sequence generation; allocation concealment; blind method; incomplete outcome data and selective reporting. Every domain could be rated as “unclear” OR “low” OR “high” risk of bias accordingly.

### Statistical analysis

RevMan5.3 software was used for statistical analysis in this meta-analysis. Continuous results were analyzed by mean difference (MD), and binary variables were evaluated by relative risk (RR). P values and 95% confidence intervals (95% CI) were also obtained. The heterogeneity between studies was tested with I^2^ statistic. When I^2^ < 50% or P > 0.1, the heterogeneity was considered acceptable, and the MD was combined according to the fixed effect model; On the contrary, if significant heterogeneity (I^2^ > 50% or P < 0.1) were considered, a random effect model was used to combine the data. Besides, we examined the robustness of meta-analysis using sensitivity analysis. P<0.05 was considered that the differences were statistically significant in this study.

## Results

### RCT selection

**Fig. 1:**
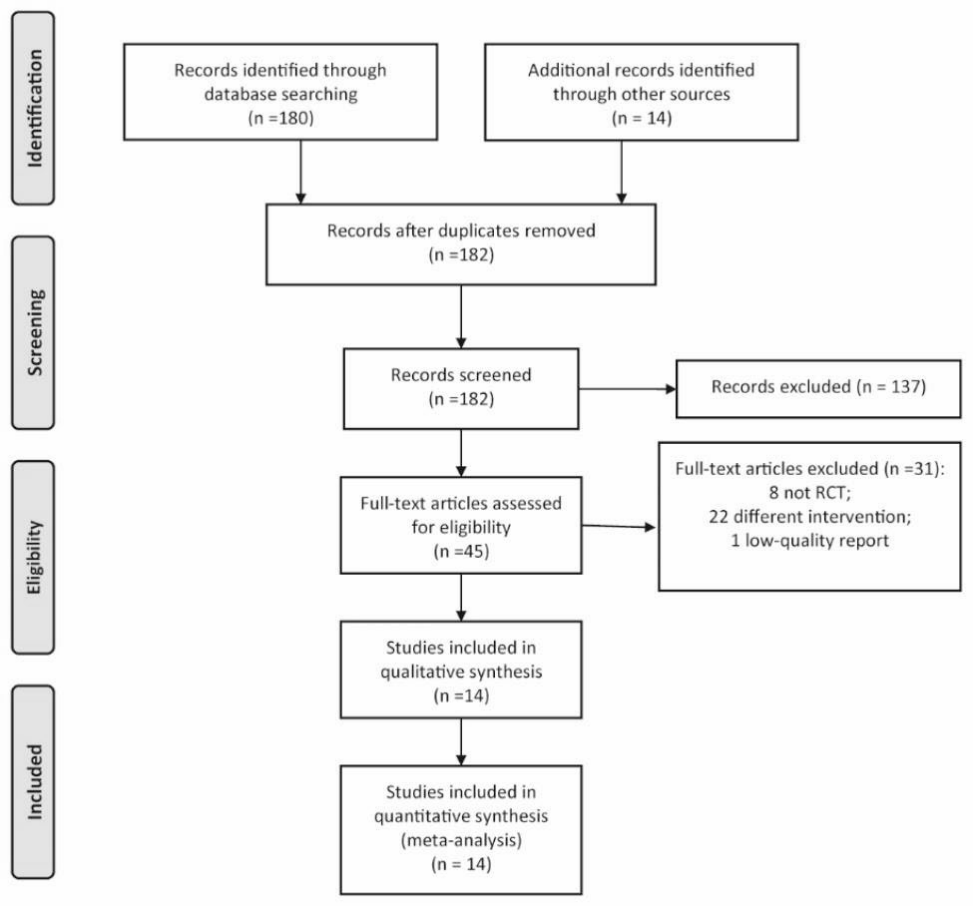
The flow chart of RCT inclusion

The process of RCT selection is presented in Fig. 1. Initially, 194 reports were identified. After removing 12 duplicates, 182 studies remained. By reviewing the title and abstract, 137 unmatched reports were further excluded. Among the remaining 45 reports, 31 studies were removed after reading the full text. Finally, 14 RCTs [13–26] were included in this meta-analysis.

### The characteristics of included RCTs

The 14 included RCTs were published between 2012 and 2020. A total of 2058 children were included in this meta-analysis, including 995 children in the orthokeratology group and 1063 children in the control group. All the included RCTs reported that there were no significant differences in the age, gender et al. characteristics. The characteristics of the included RCTs are presented in Table 1.

**Table 1:** The characteristics of included RCTs

RCT	Country	Sample size		Number of eyes		Age(years)		Criteria			Type of orthokeratology		Wearing method	Duration of follow-up(year)		
		Orthokeratology group	Control group	Orthokeratology group	Control group											
Bian 2020	China	100	100	100	100	8 ~ 14		Diopter: -0.75~-5.00D; Cis return astigmatism < 1.50D			IV-DF Dream David		Night wear	1		
Charm 2013	China	12	16	24	32	8 ~ 16		SEQ refraction at least − 5.75 diopters (D) and myopia − 5.00 D			NA		NA	1		
Cho 2012	South Korea	37	41	74	82		6 ~ 10		Myopia degree: 0.50 ~ 4.00D; Astigmatism < 1.50D	NA		NA			2	
Dong 2013	China	123	123	236	246	8 ~ 15		Sphere scope: -0.75~-6.00 DS; Range of column mirror − 0.50~-1.50DC			Dream David		Night wear	2		
Jiang 2014	China	50	45	90	90	8 ~ 15		Spherical degree 0~-6.00 ODS; Cis return astigmatism 0~-1.50 DC			Hengtai		NA	3		
Jiang 2018	China	43	40	86	80	8 ~ 14		SEQ lens diopter: -0.15 ~ − 5.00 D; Range of cis return astigmatism < 150 D			Hengtai		Night wear	2		
Li 2016	China	48	48	96	96	8 ~ 18		Diopter: -0.15D~-6.00D; Conforming astigmatism < 1.50 D			NA		NA	1		
Li 2020	China	75	75	150	150	12 ~ 18		Diopter: -2.00~-4.30 DS, − 0.50~-1.00 DC			Dream David		Night wear	1		
Liu 2018	USA	60	60	60	60	7 ~ 12		Diopter: − 3.50~-6.00 D; Astigmatism<-1.50D			Jingshi Optics		Night wear	1		
Lv 2019	China	60	62	60	62	6–15		Diopter: − 2.00~-5.0D; Astigmatism<-2.50D			Dream David		Night wear	1		
Tang 2020	China	60	60	98	93	8 ~ 16		SEQ: − 1.50~- 6.00 D; Degree of column mirror < 1.0 0 D			NA		Night wear	1		
Zhang 2017	China	80	80	160	160	12 ~ 18		Diopter: 1.00 ~ 5.00; Column mirror range ≤ -1.50 D			NA		Night wear	2		
Zhou 2016	China	167	233	334	466	9 ~ 15		Astigmatism<-1.50 DC; SEQ: -2.75 ~ 5.60 DS			Euclid		NA	2		
Zhu 2014	China	80	80	160	160	12 ~ 18		Diopter: -1.00~-4.00 DS, < 1.00 DC			NA		NA	1		

SEQ, spherical equivalent; NA, not available

### RCT quality

**Fig. 2:**
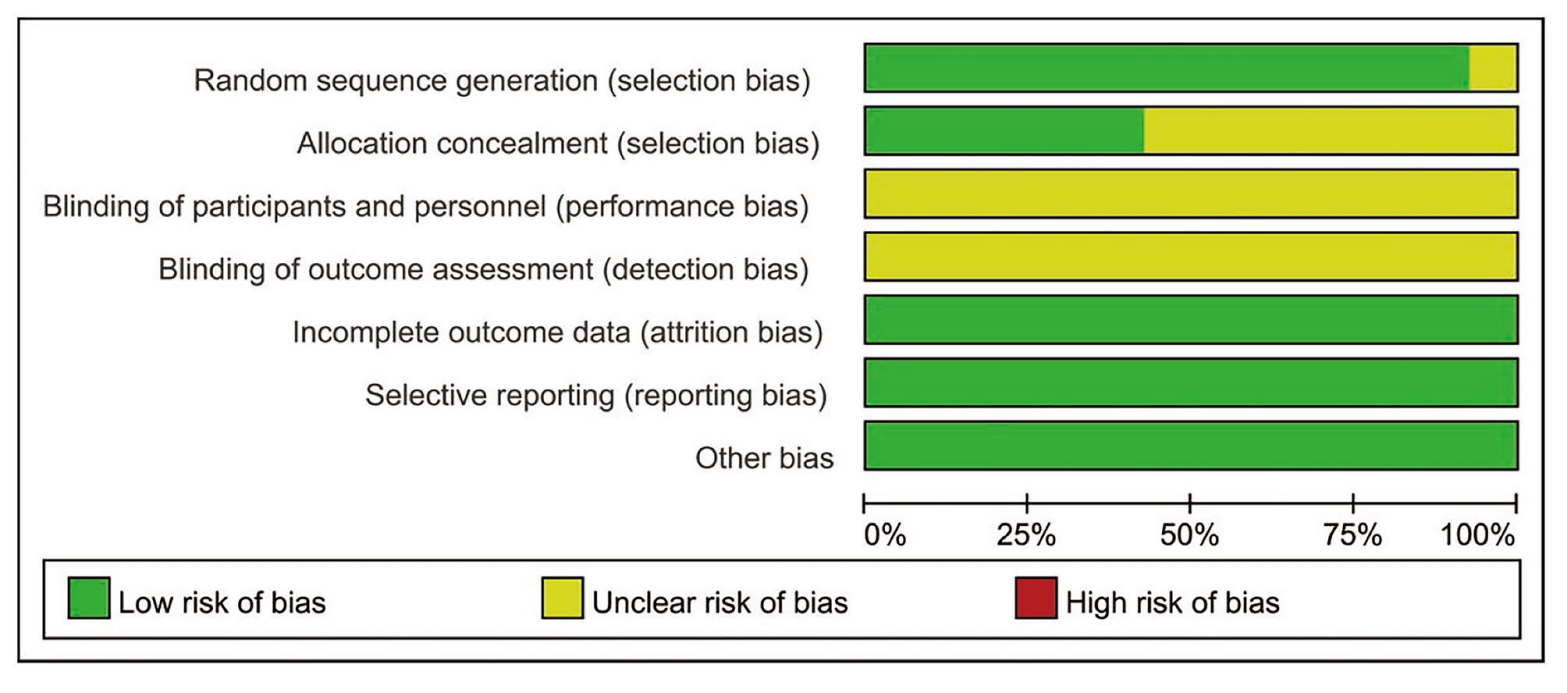
Risk of bias graph

**Fig. 3:**
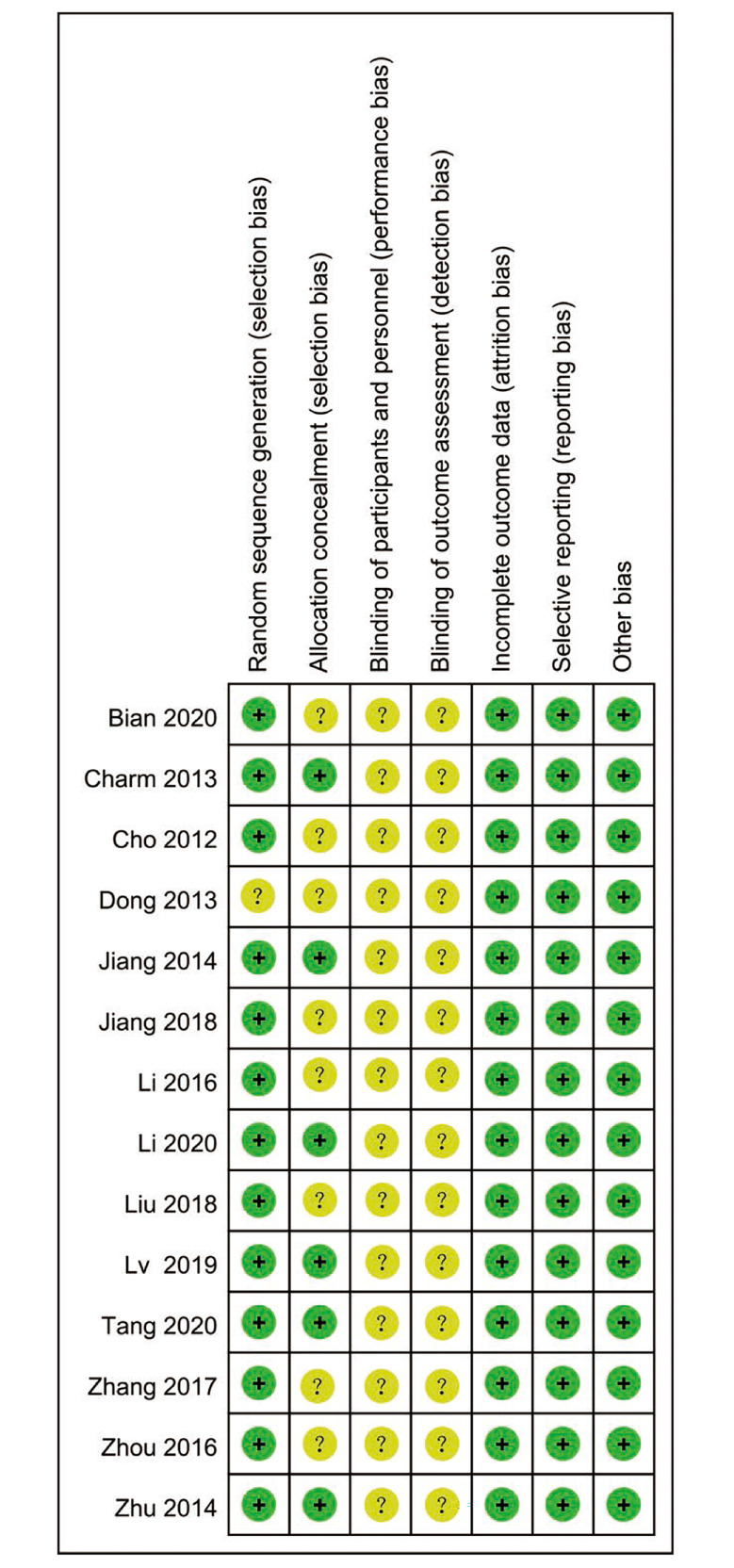
Risk of bias summary

The quality of included RCTs are showed in Figs. 2 and 3. Some studies that did not explicitly report blinding methods for intervention, outcome measurement personnel, performance bias, and detection bias were rated as ‘‘unclear”. Other evaluation items were rated as ‘‘low risk”. Generally, the included RCTs had moderate risk of bias.

### Meta-analysis

**Fig. 4:**
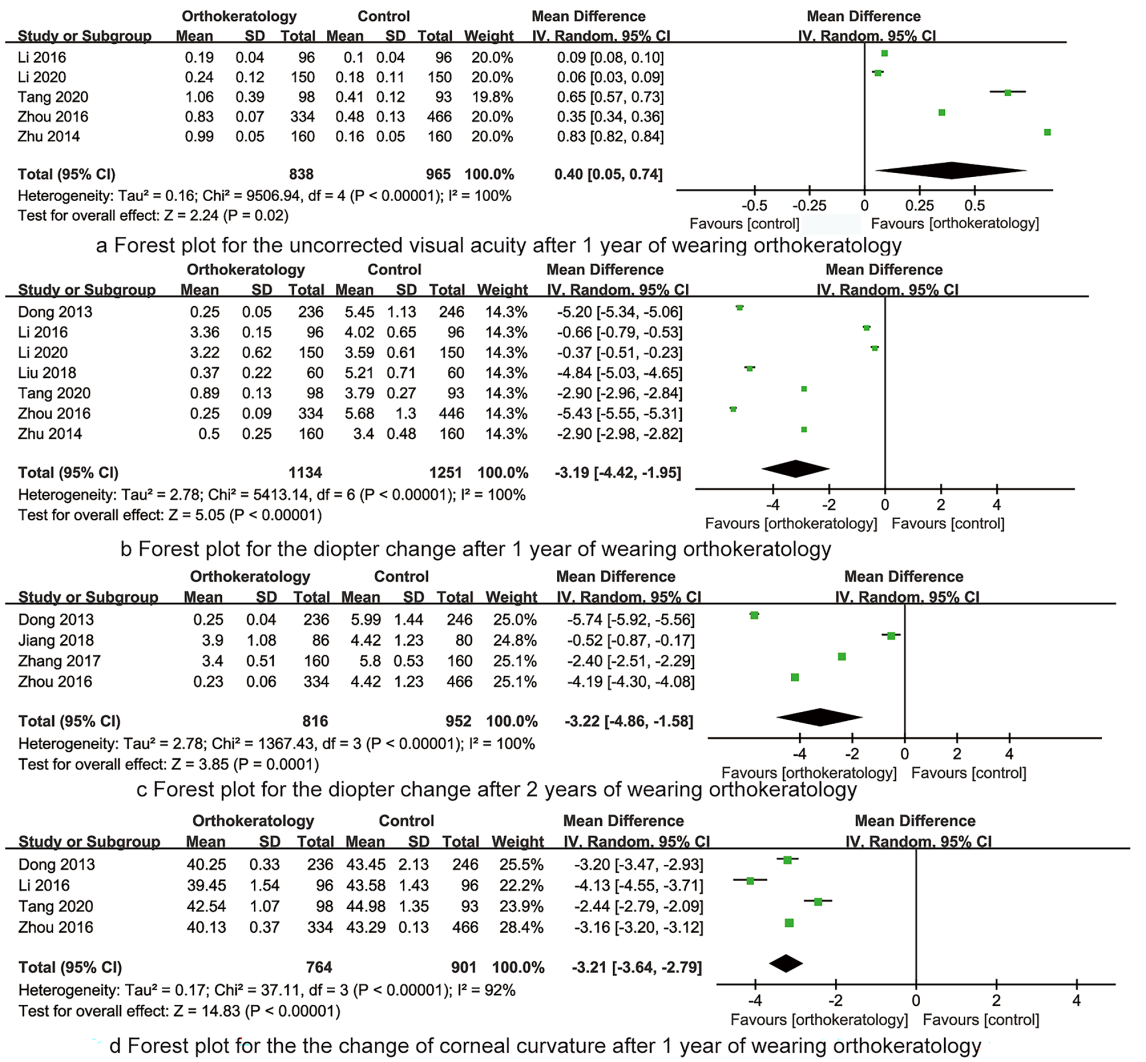
The forest plots for synthesized outcomes

*The uncorrected visual acuity after 1 year of wearing orthokeratology* Five RCTs reported the uncorrected visual acuity after 1 year of wearing orthokeratology, the result had significant heterogeneity (I^2^ = 100%, P < 0.001), then random effect model was applied for data analysis. The synthesized result indicated that the uncorrected visual acuity after 1 year of wearing orthokeratology was significantly higher than that of control group (MD = 0.40, 95%CI: 0.05 ~ 0.74, P = 0.02, Fig. 4a).

*The diopter change after 1 year of wearing orthokeratology* Seven RCTs reported the diopter change after 1 year of wearing orthokeratology, the result had significant heterogeneity (I^2^ = 100%, P < 0.001), then random effect model was applied for data analysis. The synthesized result indicated that the diopter change after 1 year of wearing orthokeratology was significantly less than that of control group (MD=-3.19, 95%CI: -4.42~-1.95, P < 0.001, Fig. 4b).

*The diopter change after 2 years of wearing orthokeratology* Four RCTs reported the diopter change after 2 year of wearing orthokeratology, the result had significant heterogeneity (I^2^ = 100%, P < 0.001), then random effect model was applied for data analysis. The synthesized result indicated that the diopter change after 2 year of wearing orthokeratology was significantly less than that of control group (MD=-3.22, 95%CI: -4.86~-1.58, P < 0.001, Fig. 4c).

*The changes of corneal curvature after 1 year of wearing orthokeratology* Four RCTs reported the changes of corneal curvature after 1 year of wearing orthokeratology, the result had significant heterogeneity (I^2^ = 92%, P < 0.001), then random effect model was applied for data analysis. The synthesized result indicated that the changes of corneal curvature after 1 year of wearing orthokeratology was significantly less than that of control group (MD=-3.21, 95%CI: -3.64~-2.79, P < 0.001, Fig. 4d).

**Fig. 5:**
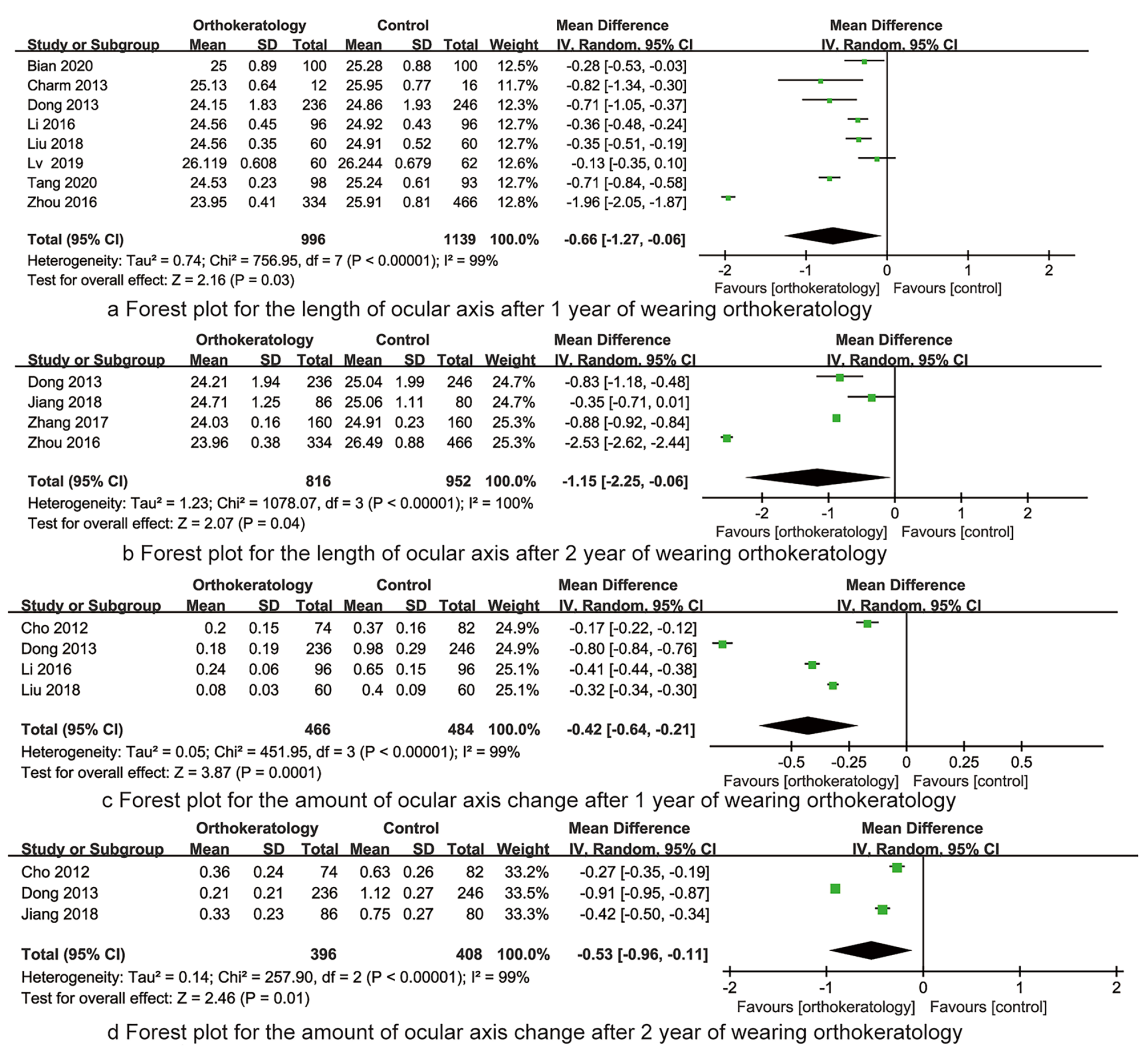
The forest plots for synthesized outcomes

*The length of ocular axis after 1 year of wearing orthokeratology* Eight RCTs reported the length of ocular axis after 1 year of wearing orthokeratology, the result had significant heterogeneity (I^2^ = 99%, P < 0.001), then random effect model was applied for data analysis. The synthesized result indicated that the length of ocular axis after 1 year of wearing orthokeratology was significantly less than that of control group (MD=-0.66, 95%CI: -1.27~-0.06, P < 0.001, Fig. 5a).

*The length of ocular axis after 2 year of wearing orthokeratology* Four RCTs reported the length of ocular axis after 2 year of wearing orthokeratology, the result had significant heterogeneity (I^2^ = 100%, P < 0.001), then random effect model was applied for data analysis. The synthesized result indicated that the length of ocular axis after 2 year of wearing orthokeratology was significantly less than that of control group (MD=-1.15, 95%CI: -2.25~-0.06, P < 0.001, Fig. 5b).

*The amount of ocular axis change after 1 year of wearing orthokeratology* Four RCTs reported the amount of ocular axis change after 1 year of wearing orthokeratology, the result had significant heterogeneity (I^2^ = 99%, P < 0.001), then random effect model was applied for data analysis. The synthesized result indicated that the amount of ocular axis change after 1 year of wearing orthokeratology was significantly less than that of control group (MD=-0.42, 95%CI: -0.64~-0.21, P < 0.001, Fig. 5c).

the amount of ocular axis change after 2 year of wearing orthokeratology Three RCTs reported the the amount of ocular axis change after 2 year of wearing orthokeratology, the result had significant heterogeneity (I^2^ = 99%, P < 0.001), then random effect model was applied for data analysis. The synthesized result indicated that the amount of ocular axis change after 2 year of wearing orthokeratology was significantly less than that of control group (MD=-0.53, 95%CI: -0.96~-0.11, P < 0.001, Fig. 5d).

### Publication bias

**Fig. 6:**
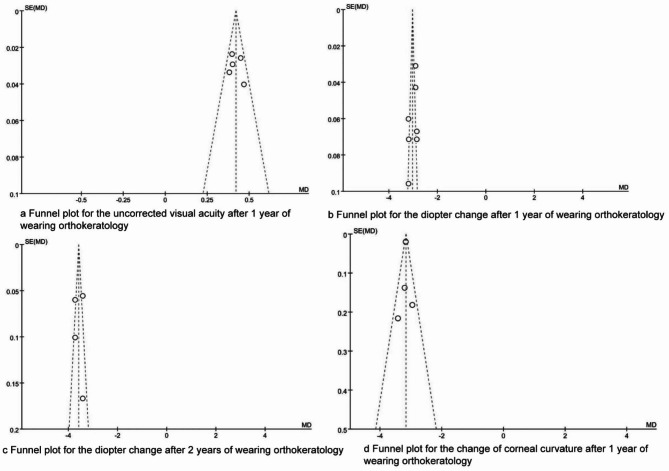
The funnel plots for synthesized outcomes

**Fig. 7:**
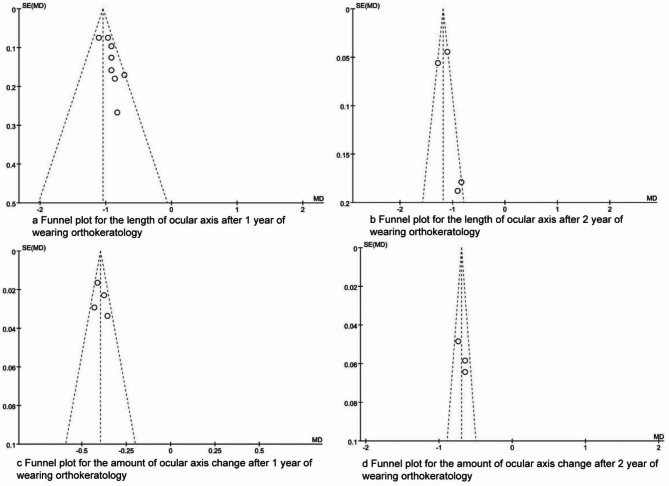
The funnel plots for synthesized outcomes

The funnel plots are presented in Figs. 6 and 7. The dots were evenly distributed in the funnel plots, and the Egger test results indicated that there were no publication biases in the synthesized outcomes (all P > 0.05).

We examined the robustness of meta-analysis using sensitivity analysis by excluding the RCTs one by one, the synthesized results did not statistically change, indicating that the synthesized outcomes were robust.

## Discussions

Myopia is a global health and social problem. The occurrence and development of the disease mainly occurs in children and adolescents. Therefore, the control of myopia has focused on children and adolescents. Myopia, especially high myopia, usually leads to serious consequences, including glaucoma, macular degeneration, retinal detachment and cataract, which may lead to irreversible visual impairment in later life [27–29].At the same time, high myopia is related to the reduction of vision related quality of life, and has a significant socio-economic impact [30, 31]. Compared with previous meta-analyses [32, 33], this study has included more sample size and analyzed outcomes. The results of this meta-analysis have showed that compared with the frame lens, the naked vision, corneal curvature, diopter, axial length and their changes of the patients with the corneal plastic lens are statistically different, and the myopia control effect are better than the frame lens. Orthokeratology is a beneficial to control the myopia progression of children, which is a good option for myopia control and care.

At present, the measures to control the progress of children’s myopia include pharmacology, environment, surgery and optics [34–36]. The drug control of myopia mainly uses atropine. 0.01% atropine can reduce refractive error by about 45%. Compared with the control group, the axial control effect is not obvious, but the side effects and reactions after drug withdrawal are less, 1% atropine may reduce myopia progression by 60%~80% [37]. The extension of outdoor activities and the reduction of children’s schoolwork burden are more effective in the primary prevention of myopia [38]. Wearing frame glasses is a common means to control myopia. However, because of the distance between the lens and the apex of the cornea, the image magnification is bound to change. The lens itself also limits the field of vision, and this phenomenon is more obvious in myopic eyes with higher degrees [39]. Compared with frame glasses, Orthokeratology has unique advantages. The distance between the lens and the eye is reduced, it can minimize the magnification reduction of the retinal image caused by high refraction [38, 40]. However, it must be noted that there is a lack of corneal topography analysis after orthokeratology to define the optical effect of the molding on peripheral defocus from each study. Contrary to soft multifocal or glasses, the visual impact on peripheral defocus of orthokeratology can vary with the brand and the fitter philosophy.

Myopia is the result of genetic and environmental factors, and its pathogenesis and progression mechanism are still unclear. At present, the mainstream mechanisms include regulation mechanism, hyperopic defocusing mechanism, etc [41, 42]. The stimulating effect of periretinal hyperopia defocus on central axial myopia [43]. It has been reported that in the later stage, significant differences in axial length and peripheral retinal morphology are found between people with progressive myopia and those with stable myopia [44]. Previous research [45] shows that myopic defocusing can slow down the progress of myopia. The principle of orthokeratology to control myopia is based on defocusing theory. Compared with the traditional monocular frame glasses that may increase the peripheral hyperopia defocusing, orthokeratology changes the central shape of the cornea, promotes the migration of corneal epithelial cells, inhibits hyperopia defocusing, and provides myopia defocusing for the peripheral retina through the mechanical pressure of the flat base arc designed in inverse geometry and the negative pressure suction of the tear under the reverse arc [46, 47]. For astigmatic patients, the progression of myopia is not related to the initial astigmatism, but related to the way of myopia control [48]. Therefore, the rational use of orthokeratology can effectively control the development of myopia in children.

It’s been reported that the intraocular pressure measured by non-contact intraocular pressure after orthokeratology is lower than the actual value, and is significantly related to the thinning of central corneal thickness after wearing glasses [49]. Previous study [50] has measured intraocular pressure with dynamic contour tonometer before and after wearing, there is no significant difference. They have believed that there is no effect on actual intraocular pressure after orthokeratology. Myopia is one of the risk factors of glaucoma. For patients who use non-contact tonometer to recheck intraocular pressure, they may miss the early stage of glaucoma, so they should be alert in clinical work [51]. Previous study [52] has found that Goldmann related intraocular pressure and corneal compensated intraocular pressure decreased one week after orthokeratology, and have become stable after reaching the minimum one week. The mechanism of this decrease in intraocular pressure may be that the base arc of the lens contacts the center of the friction cornea, and the compression force of the eyelids produces a continuous massage force on the eyeball, forcing the aqueous humor to drain faster, so that the intraocular pressure decreases [53]. Therefore, orthokeratology is a safe means to prevent and control myopia, but improving the visual quality of the lens optical area, reducing corneal irritation, improving tear circulation and tear film stability are the improvement directions of orthokeratology [54–56]. For myopic patients, it is very necessary to follow up regularly and strengthen lens care.

There are some limitations in this study that are worth considering. Firstly, fewer high-quality documents are included, and the possibility of bias and error is increased; Secondly, there is too large I^2^ amongst the results, yet we can not perform the subgroup analysis limited by the reported data. Thirdly, we only include Chinese and English literature, which may have some language bias; Finally, the number of included RCTs and the sample size were limited, and extrapolation of the meta-analysis results was limited to some extent. Future studies with rigorous design from different areas are needed to evaluate the effects of orthokeratology in myopia control.

## Conclusions

In conclusion, existing evidences have showed that orthokeratology has a positive effect on slowing down the development of myopia in children. Whether there are differences in the long-term efficacy and safety of OK for children of different ethnic groups, as well as the efficacy of different OK for patients with different myopia and different ages, still needs to be further verified by a large sample of high-quality RCTs.

## Data Availability

All data generated or analyzed during this study are included in this published article.

## Abbreviations

RCTs: Randomized controlled trials
PRISMA: Preferred reporting items for systematic reviews and meta-analyses
MD: Mean difference
RR: Relative risk
CI: Confidence interval

## References

[1] Han X, Liu C, Chen Y, He M (2022). Myopia prediction: a systematic review. Eye (Lond).

[2] Alvarez-Peregrina C, Martinez-Perez C, Sanchez-Tena MA. Myopia and other Visual disorders in Children. Int J Environ Res Public Health 2022, 19(15). 10.3390/ijerph19158912 PMC9332575 35897282

[3] Hiraoka T (2022). Myopia control with Orthokeratology: a review. Eye Contact Lens.

[4] Tricard D, Marillet S, Ingrand P, Bullimore MA, Bourne RRA, Leveziel N (2022). Progression of myopia in children and teenagers: a nationwide longitudinal study. Br J Ophthalmol.

[5] Russo A, Boldini A, Romano D, Mazza G, Bignotti S, Morescalchi F, Semeraro F (2022). Myopia: mechanisms and strategies to slow down its progression. J Ophthalmol.

[6] Lee SS, Lingham G, Sanfilippo PG, Hammond CJ, Saw SM, Guggenheim JA, Yazar S, Mackey DA (2022). Incidence and progression of myopia in early adulthood. JAMA Ophthalmol.

[7] Wang Y, Liu Y, Zhu X, Zhou X, He JC, Qu X. Corneal and lenticular biometry in Chinese children with myopia. Clin Exp Optom 2022:1–9. 10.1080/08164622.2022.2116269 36045001

[8] Wang YM, Lu SY, Zhang XJ, Chen LJ, Pang CP, Yam JC. Myopia Genetics and Heredity. Child (Basel) 2022, 9(3). 10.3390/children9030382 PMC8947159 35327754

[9] Matsumura S, Dannoue K, Kawakami M, Uemura K, Kameyama A, Takei A, Hori Y (2022). Prevalence of myopia and its Associated factors among Japanese Preschool Children. Front Public Health.

[10] Dragomirova M, Antonova A, Stoykova S, Mihova G, Grigorova D (2022). Myopia in Bulgarian school children: prevalence, risk factors, and health care coverage. BMC Ophthalmol.

[11] Page MJ, McKenzie JE, Bossuyt PM, Boutron I, Hoffmann TC, Mulrow CD, Shamseer L, Tetzlaff JM, Akl EA, Brennan SE (2021). The PRISMA 2020 statement: an updated guideline for reporting systematic reviews. BMJ.

[12] Higgins JP, Altman DG, Gotzsche PC, Juni P, Moher D, Oxman AD, Savovic J, Schulz KF, Weeks L, Sterne JA (2011). The Cochrane collaboration’s tool for assessing risk of bias in randomised trials. BMJ.

[13] Bian S, Liu H, Lin J (2020). A randomized controlled study on the clinical effect of myopia children’s corneal plastic lens and frame glasses for one year. Chin J Experimental Ophthalmol.

[14] Charm J, Cho P (2013). High myopia-partial reduction ortho-k: a 2-year randomized study. Optom Vis Sci.

[15] Cho P, Cheung SW (2012). Retardation of myopia in Orthokeratology (ROMIO) study: a 2-year randomized clinical trial. Invest Ophthalmol Vis Sci.

[16] Dong J, Liu Z, Feng Y (2013). A comparative study on the correction of juvenile myopia with orthokeratology and ordinary frame glasses. Chin J Strabismus Pediatr Ophthalmol.

[17] Jiang D, Li M, Cao S (2014). Long term effect study of orthokeratology on myopia correction in adolescents. J Hubei Univ Sci Technol.

[18] Jiang L, Zhu Y, Zhang Y (2018). Clinical observation on the correction of low to moderate myopia with corneal plastic lens. China Grass Roots Medicine.

[19] Li W (2020). Clinical study on Orthokeratology Controlling the Progress of Myopia in adolescents. Health.

[20] Li Y (2016). Corneal plastic lenses in controlling the development of juvenile myopia.

[21] Liu C, Xin X (2018). Short term clinical effect of primary school students wearing orthokeratology. J Baotou Med Coll.

[22] Lv T, Wang L, Zhou L, Qin J, Ma H, Shi M (2020). Regimen study of high myopia-partial reduction Orthokeratology. Eye Contact Lens.

[23] Tang H, Ling H, Wu X (2020). Monitoring the role of orthokeratology in the prevention and control of juvenile myopia through ocular axis measurement. China Contemp Med.

[24] Zhang C, Wei M (2017). Clinical observation on the treatment of juvenile mild to moderate myopia with orthokeratology. Disease Monit Control.

[25] Zhou Z, Xu S, Yi S (2016). Curative effect of orthokeratology on juvenile myopia astigmatism and its influence on corneal endothelial cells. Int J Ophthalmol.

[26] Zhu F (2014). Clinical study on the control of juvenile myopia progress by orthokeratology.

[27] Rathi M, Chhabra S, Sachdeva S, Rustagi IM, Soni D, Dhania S (2022). Correlation of parental and childhood myopia in children aged 5–16 years in North India. Indian J Ophthalmol.

[28] Ovenseri-Ogbomo G, Osuagwu UL, Ekpenyong BN, Agho K, Ekure E, Ndep AO, Ocansey S, Mashige KP, Naidoo KS, Ogbuehi KC (2022). Systematic review and meta-analysis of myopia prevalence in African school children. PLoS ONE.

[29] Lipson MJ, Boland B, McAlinden C (2022). Vision-related quality of life with myopia management: a review. Cont Lens Anterior Eye.

[30] Zhang J, Li Z, Ren J, Wang W, Dai J, Li C, Huang X, Sun X, Liu L, Wang C (2022). Prevalence of myopia: a large-scale population-based study among children and adolescents in weifang, China. Front Public Health.

[31] Zhang H, Lam CSY, Tang WC, Leung M, Qi H, Lee PH, To CH. Myopia control effect is influenced by baseline relative peripheral refraction in children wearing Defocus Incorporated multiple segments (DIMS) spectacle lenses. J Clin Med 2022, 11(9). 10.3390/jcm11092294 PMC9099701 35566423

[32] Guan M, Zhao W, Geng Y, Zhang Y, Ma J, Chen Z, Peng M, Li Y (2020). Changes in axial length after orthokeratology lens treatment for myopia: a meta-analysis. Int Ophthalmol.

[33] Tsai HR, Wang JH, Huang HK, Chen TL, Chen PW, Chiu CJ (2022). Efficacy of atropine, orthokeratology, and combined atropine with orthokeratology for childhood myopia: a systematic review and network meta-analysis. J Formos Med Assoc.

[34] Liu YL, Jhang JP, Hsiao CK, Tsai TH, Wang IJ (2022). Influence of parental behavior on myopigenic behaviors and risk of myopia: analysis of nationwide survey data in children aged 3 to 18 years. BMC Public Health.

[35] Wang W, Xiang Y, Zhu L, Zheng S, Ji Y, Lv B, Xiong L, Li Z, Yi S, Huang H (2022). Myopia progression and associated factors of refractive status in children and adolescents in Tibet and Chongqing during the COVID-19 pandemic. Front Public Health.

[36] Gopalakrishnan A, Hussaindeen JR, Sivaraman V, Swaminathan M, Wong YL, Armitage JA, Gentle A, Backhouse S (2022). Prevalence of myopia among urban and suburban school children in Tamil Nadu, South India: findings from the Sankara Nethralaya Tamil Nadu Essilor Myopia (STEM) study. Ophthalmic Physiol Opt.

[37] Zhu L, Ding L, Wang J (2022). Meta analysis of the effectiveness and safety of corneal plastic lens in controlling myopia in primary and secondary school students. Chin School Doctor.

[38] Zhang KY, Lyu HB, Yang JR, Qiu WQ (2022). Efficacy of long-term orthokeratology treatment in children with anisometropic myopia. Int J Ophthalmol.

[39] Cho P, Cheung SW (2017). Protective role of Orthokeratology in reducing risk of Rapid Axial Elongation: a reanalysis of Data from the ROMIO and TO-SEE studies. Invest Ophthalmol Vis Sci.

[40] Lipson MJ (2022). The role of Orthokeratology in Myopia Management. Eye Contact Lens.

[41] Tao Z, Wang J, Zhu M, Lin Z, Zhao J, Tang Y, Deng H (2021). Does Orthokeratology wearing affect the tear quality of children?. Front Pediatr.

[42] Chen X, Xiong Y, Liu F, Wang J, Yang B, Liu L (2022). Factors determining the myopia control effect of an orthokeratology lens: a two-year multi-level model. Ophthalmic Physiol Opt.

[43] Shinojima A, Negishi K, Tsubota K, Kurihara T (2022). Multiple factors causing myopia and the possible treatments: a Mini Review. Front Public Health.

[44] Jiang M, Zhou Q, Chen J, Liu X, Wang L (2014). Meta analysis of the influence of corneal plastic lens on the ocular axis of juvenile myopia. Chin J Practical Ophthalmol.

[45] Guan D, Xu J (2018). Analysis of the efficacy and safety of corneal plastic lens in controlling juvenile myopia. Gansu Med.

[46] Duan C, Feng F, Liu L, Qu F, Yang Z, Zhang H, Jiang C (2022). Group-based trajectory modeling to identify factors influencing the development of myopia in patients receiving Orthokeratology. Int J Gen Med.

[47] Sun L, Li ZX, Chen Y, He ZQ, Song HX (2022). The effect of orthokeratology treatment zone decentration on myopia progression. BMC Ophthalmol.

[48] Tang WT, Li SB, Li YJ, Tang ZP, Ma D (2022). [Clinical observation of orthokeratology with increased compression factor in the treatment of myopia]. Zhonghua Yan Ke Za Zhi.

[49] Huo S, Liu B, Zhou S (2019). Research progress on the safety and effectiveness of keratoplasty for myopia. J Local Surg.

[50] Dapeng T (2018). Current status of research on the efficacy and safety of corneal plastic lens in the treatment of myopia. Practical Clin Med.

[51] Rui W, Jingru S, Xiangxia L (2019). Research progress in the correction of myopia with corneal plastic lens. J Eye Ear Nose Throat Traditional Chin Med.

[52] Jun J, Xiaomei Q, Xiao Y (2020). Effectiveness and safety of aspherical keratoplasty lens in correcting myopia. Chin J Optometry Visual Sci.

[53] Meijuan Y, Quan L (2022). Research progress in the control of myopia anisometropia with corneal plastic lens. Int J Ophthalmol.

[54] Kuo YK, Chen YT, Chen HM, Wu PC, Sun CC, Yeung L, Lin KK, Chen HC, Chuang LH, Lai CC et al. Efficacy of myopia control and distribution of corneal epithelial thickness in children treated with Orthokeratology assessed using Optical Coherence Tomography. J Pers Med 2022, 12(2). 10.3390/jpm12020278 PMC8875657 35207766

[55] Ding C, Chen Y, Li X, Huang Y, Chen H, Bao J (2022). The associations of accommodation and aberrations in myopia control with orthokeratology. Ophthalmic Physiol Opt.

[56] Jakobsen TM, Moller F (2022). Control of myopia using orthokeratology lenses in scandinavian children aged 6 to 12 years. Eighteen-month data from the Danish Randomized Study: clinical study of Near-sightedness; TReatment with orthokeratology lenses (CONTROL study). Acta Ophthalmol.

